# Supplementary material for: Elucidating the impact of Hypericum alpestre extract and L-NAME on the PI3K/Akt signaling pathway in A549 lung adenocarcinoma and MDA-MB-231 triple-negative breast cancer cells
Source: PLoS One. 2025 Apr 3;20(4):e0303736. doi: 10.1371/journal.pone.0303736 (PMC11967956; doi:10.1371/journal.pone.0303736)
Supplement: S1 Table — (DOCX) [file pone.0303736.s001.docx]

**Suppl. Table. Phytochemicals tentatively identified by LC-Q-Orbitrap-HRMS in *H. alpestre* aerial ethanol part extract**

| **Peak no.** | **Compound Group** | **Tentative Identification** | **RT [min]** | **Molecular Formula** | **Molecular Weight** | **Theoretical (m/z)** | **Observed (m/z)** | **Mass Error (ppm)** | **Fragments (m/z)** |
| --- | --- | --- | --- | --- | --- | --- | --- | --- | --- |
| 1 | COA | Gluconic acid | 1.80 | C_6_H_12_O_7_ | 196.05831 | 195.05048 | 195.05013 | 1.82 | - |
| 2 | CA | Quinic acid | 1.85 | C_7_H_12_O_6_ | 192.06339 | 191.05557 | 191.05529 | 1.47 | 85.02 |
| 3 | HT | Galloyl glucose isomer | 2.84 | C_13_H_16_O_10_ | 332.07435 | 331.06653 | 331.06705 | -1.57 | 125.02; 151.00; 169.01 |
| 4 | HBA | Salicylic acid glucoside | 3.18 | C_13_H_16_O_8_ | 300.08452 | 299.07670 | 299.07717 | -1.57 | 93.03; 137.02 |
| 5 | HT | Galloyl glucose isomer | 3.65 | C_13_H_16_O_10_ | 332.07435 | 331.06653 | 331.06712 | -1.78 | 125.02; 169.01 |
| 6 | HT | Vanillic acid glucoside | 3.86 | C_14_H_18_O_9_ | 330.09509 | 329.08726 | 329.08776 | -1.50 | 108.02; 152.01; 167.03 |
| 7 | F-3-ol | Gallocatechin | 4.59 | C_15_H_14_O_7_ | 306.07396 | 305.06613 | 305.06664 | -1.66 | 125.02; 139.04; 167.03 |
| 8 | PG | Hypericophenonoside | 4.89 | C_19_H_20_O_11_ | 424.10057 | 423.09274 | 423.09354 | -1.88 | 151.00; 261.04 |
| 9 | HCA | Caffeoylquinic acid isomer | 5.17 | C_16_H_18_O_9_ | 354.09509 | 353.08726 | 353.08760 | -0.95 | 135.04; 179.03; 191.05 |
| 10 | HCA | Caffeoyl glucose isomer | 5.18 | C_15_H_18_O_9_ | 342.09509 | 341.08726 | 341.08760 | -0.98 | 135.04; 179.03 |
| 11 | PG | Phaseoloidin | 5.32 | C_14_H_18_O_9_ | 330.09509 | 329.08726 | 329.08772 | -1.38 | 123.04; 149.02; 167.03 |
| 12 | CT | B-type procyanidin dimer | 5.34 | C_30_H_26_O_13_ | 594.13735 | 593.12952 | 593.13038 | -1.44 | 289.07; 407.08 |
| 13 | HCA | Coumaroyl glucose isomer | 5.36 | C_15_H_18_O_8_ | 326.10017 | 325.09235 | 325.09279 | -1.35 | 119.05; 163.04 |
| 14 | PG | Salidroside | 5.39 | C_14_H_20_O_7_ | 300.12091 | 299.11308 | 299.11359 | -1.69 | 119.04; 137.02 |
| 15 | F-3-ol | Epi/catechin hexoside | 5.52 | C_21_H_24_O_11_ | 452.13187 | 451.12404 | 451.12488 | -1.85 | 245.08; 289.07 |
| 16 | HCA | Caffeoyl glucose isomer | 5.61 | C_15_H_18_O_9_ | 342.09509 | 341.08726 | 341.08762 | -1.04 | 161.02; 179.03 |
| 17 | PG | Dihydroxybenzoic acid hexoside pentoside | 5.66 | C_19_H_28_O_12_ | 448.15808 | 447.15026 | 447.15101 | -1.68 | 109.03; 153.02 |
| 18 | FA | Glucosyl-hexanoic acid isomer | 5.66 | C_12_H_22_O_8_ | 294.13147 | 293.12365 | 293.12418 | -1.81 | 59.01; 71.01; 89.02; 113.07; 131.07 |
| 19 | CT | B-type procyanidin dimer | 5.96 | C_30_H_26_O_12_ | 578.14243 | 577.13461 | 577.13547 | -1.49 | 125.02; 289.07; 407.08 |
| 20 | PC | Ethylvanillin glucoside | 6.16 | C_15_H_20_O_8_ | 328.11582 | 327.10800 | 327.10856 | -1.71 | 59.01; 121.06; 165.05 |
| 21 | F-3-ol | Gallocatechin | 6.23 | C_15_H_14_O_7_ | 306.07396 | 305.06613 | 305.06659 | -1.49 | 125.02; 139.04; 167.03 |
| 22 | HCA | Feruloyl glucose isomer | 6.29 | C_16_H_20_O_9_ | 356.11074 | 355.10291 | 355.10345 | -1.51 | 134.03; 193.05 |
| 23 | HBA | Hydroxybenzoic acid glucoside | 6.39 | C_13_H_16_O_8_ | 300.08452 | 299.07670 | 299.07718 | -1.60 | 93.03; 137.02 |
| 24 | CT | Procyanidin tetramer one A-type linkage | 6.58 | C_60_H_48_O_24_ | 1152.25356 | 1151.24574 | 1151.24730 | -1.36 | 289.07; 711.13; 863.11; |
| 25 | HCA | p-Coumaroylquinic acid | 6.92 | C_16_H_18_O_8_ | 338.10017 | 337.09235 | 337.09278 | -1.28 | 119.05; 163.04; 191.06 |
| 26 | F-3-ol | B-type procyanidin dimer | 6.92 | C_30_H_26_O_12_ | 578.14243 | 577.13461 | 577.13533 | -1.25 | 125.02; 289.07; 407.08 |
| 27 | FO | Quercetin-glucosyl-xyloside isomer | 7.02 | C_26_H28O16 | 596.13774 | 595.12992 | 595.13062 | -1.18 | 301.04; 462.08 |
| 28 | CT | Procyanidin tetramer | 7.03 | C_60_H_50_O_24_ | 1154.26921 | 1153.26139 | 1153.26284 | -1.26 | 575.12; 866.19 |
| 29 | HCA | Sinapoyl-glucoside | 7.07 | C_17_H_22_O_10_ | 386.12130 | 385.11348 | 385.11417 | -1.79 | 164.05; 223.06 |
| 30 | FA | Glucosyl-hexanoic acid isomer | 7.13 | C_12_H_22_O_8_ | 294.13147 | 293.12365 | 293.12421 | -1.91 | 59.01; 71.01; 89.02; 131.07 |
| 31 |  | Unknown | 7.13 | C_17_H_30_O_13_ | 442.16865 | 441.16082 | 441.16149 | -1.51 | 89.02; 101.06 |
| 32 | HCA | Coumaroyl-glucose isomer | 7.20 | C_15_H_18_O_8_ | 326.10017 | 325.09235 | 325.09284 | -1.51 | 119.05; 163.04 |
| 33 | CT | Procyanidin trimer | 7.39 | C_45_H_38_O_18_ | 866.20582 | 865.19800 | 865.19920 | -1.39 | 287.06; 425.08; 577.14; 695.14 |
| 34 | HCA | Caffeoylquinic acid isomer | 7.45 | C_16_H_18_O_9_ | 354.09509 | 353.08726 | 353.08753 | -0.75 | 135.04; 173.04; 191.05 |
| 35 |  | Unknown | 7.46 | C_14_H_22_O_9_ | 334.12639 | 333.11856 | 333.11902 | -1.37 | 127.07 |
| 36 | FA | Tuberonic acid glucoside | 7.52 | C_18_H_28_O_9_ | 388.17334 | 387.16551 | 387.16590 | -0.99 | 163.11 |
| 37 | F-3-ol | Catechin | 7.54 | C_15_H_14_O_6_ | 290.07904 | 289.07122 | 289.07171 | -1.70 | 109.03; 123.04; 125.02; 151.04 |
| 38 | HCA | Caffeoylquinic acid methyl ester | 7.71 | C_17_H_20_O_9_ | 368.11074 | 367.10291 | 367.10331 | -1.08 | 134.04; 193.05 |
| 39 |  | Unknown | 7.97 | C18H32O13 | 456.18430 | 455.17647 | 455.17719 | -1.57 | 101.06; 247.15 |
| 40 | PG | Phaseoloidin | 8.00 | C_14_H_18_O_9_ | 330.09509 | 329.08726 | 329.08775 | -1.47 | 123.04; 167.03 |
| 41 | CT | Procyanidin pentamer one A-type linkage | 8.08 | C_75_H_60_O_30_ | 1440.31695 | 1439.30913 | 1439.31150 | -1.65 | 719.15 [M-2H]^-2^ (289.07; 411.07; 693.13) |
| 42 |  | Unknown | 8.14 | C_20_H_32_O_10_ | 432.19955 | 431.19173 | 431.19225 | -1.21 | 89.02 |
| 43 | HCA | Feruloyl glucose isomer | 8.17 | C_16_H_20_O_9_ | 356.11074 | 355.10291 | 355.10340 | -1.37 | 134.03; 178.03; 193.05 |
| 44 | FO | Quercetin-glucosyl-xyloside isomer | 8.23 | C_26_H_28_O_16_ | 596.13774 | 595.12992 | 595.13062 | -1.18 | 301.04; 463.08 |
| 45 |  | Unknown | 8.26 | C_13_H_14_O_6_ | 266.07904 | 265.07122 | 265.07172 | -1.89 | 117.03; 145.03 |
| 46 | CT | B-type procyanidin dimer | 8.40 | C_30_H_26_O_12_ | 578.14243 | 577.13461 | 577.13531 | -1.21 | 125.02; 289.07; 407.08 |
| 47 | PC | Ethylvanillin glucoside | 8.63 | C_15_H_20_O_8_ | 328.11582 | 327.10800 | 327.10845 | -1.38 | 147.04; 165.05 |
| 48 | FN | Apigenin -methyl glucuronate | 8.70 | C_20_H_30_O_12_ | 462.17373 | 461.16591 | 461.16660 | -1.50 | 101.02; 161.04; 269.10 |
| 49 |  | Unknown | 8.71 | C_19_H_30_O_8_ | 386.19407 | 385.18625 | 385.18670 | -1.17 | 259.06; 271.06; 365.17 |
| 50 |  | Unknown | 8.72 | C_20_H_32_O_10_ | 432.19955 | 431.19173 | 431.19236 | -1.46 | 285.04 |
| 51 |  | Unknown | 8.72 | C_27_H_38_O_12_ | 586.22616 | 585.21834 | 585.21915 | -1.38 | 195.07; 329.14; 377.16 |
| 52 | CT | Procyanidin tetramer | 8.74 | C_60_H_50_O_24_ | 1154.26921 | 1153.26139 | 1153.26293 | -1.34 | 287.06; 575.12; 865.20; 983.20 |
| 53 | BG | Tetrahydroxybenzophenon glucuronide | 8.82 | C_19_H_18_O_11_ | 422.08492 | 421.07709 | 421.07761 | -1.22 | 245.04; 255.09 |
| 54 |  | Unknown | 8.83 | C_13_H_24_O_8_ | 308.14712 | 307.13930 | 307.13965 | -1.14 | 59.01; 127.07 |
| 55 | BG | Pentahydroxybenzophenone xylosyl-rutinoside | 8.89 | C_31_H_42_O_18_ | 702.23712 | 701.22930 | 701.23017 | -1.24 | 261.04; 393.08 |
| 56 | BG | Pentahydroxybenzophenone arabinopyranoside | 8.93 | C_18_H_18_O_10_ | 394.09000 | 393.08218 | 393.08280 | -1.58 | 243.03; 261.04 |
| 57 | HCA | Coumaroyl-glucose isomer | 9.01 | C_15_H_18_O_8_ | 326.10017 | 325.09235 | 325.09287 | -1.60 | 145.03; 163.04 |
| 58 | XD | Mangiferin | 9.10 | C_19_H_18_O_11_ | 422.08492 | 421.07709 | 421.07761 | -1.22 | 259.02; 301.04; 331.05 |
| 59 | F-3-ol | Epicatechin | 9.30 | C_15_H_14_O_6_ | 290.07904 | 289.07122 | 289.07163 | -1.42 | 109.03; 123.04; 125.02; 151.04 |
| 60 | HCA | p-Coumaroylquinic acid isomer | 9.57 | C_16_H_18_O_8_ | 338.10017 | 337.09235 | 337.09287 | -1.54 | 119.05; 163.04; 173.04; 191.06 |
| 61 | FNN | Taxifolin-rhamnoside | 9.65 | C_21_H_22_O_11_ | 450.11622 | 449.10839 | 449.10912 | -1.61 | 178.99; 259.06; 287.06 |
| 62 |  | Unknown | 9.85 | C_13_H_24_O_8_ | 308.14712 | 307.13930 | 307.13983 | -1.73 | 59.01; 127.07 |
| 63 | FO | Quercetin-diglucoside isomer | 9.88 | C_27_H_30_O_17_ | 626.14831 | 625.14048 | 625.14136 | -1.40 | 301.04; 463.09 |
| 64 | CT | Procyanidin trimer one A-type linkage | 9.93 | C_45_H_36_O_18_ | 864.19017 | 863.18235 | 863.18375 | -1.62 | 289.07; 411.07; 451.10; 573.10; 711.13 |
| 65 | CT | Procyanidin trimer | 9.95 | C_45_H_38_O_18_ | 866.20582 | 865.19800 | 865.19772 | 0.32 | 287.06; 425.08; 577.14; 695.14 |
| 66 |  | Unknown | 10.05 | C_20_H_30_O_12_ | 462.17373 | 461.16591 | 461.16669 | -1.69 | 89.02; 149.04; 167.03; 191.06 |
| 67 | HCA | Caffeoylquinic acid methyl ester | 10.36 | C_17_H_20_O_9_ | 368.11074 | 367.10291 | 367.10337 | -1.24 | 134.04; 173.04; 193.05 |
| 68 |  | Unknown | 10.38 | C_26_H_34_O_13_ | 554.19995 | 553.19212 | 553.19306 | -1.69 | 327.12; 477.17 |
| 69 | CT | Procyanidin tetramer one A-type linkage | 10.40 | C_60_H_48_O_24_ | 1152.25356 | 1151.24574 | 1151.24756 | -1.58 | 289.07; 711.13; 863.11; |
| 70 | CT | Procyanidin tetramer | 10.55 | C_60_H_50_O_24_ | 1154.26921 | 1153.26139 | 1153.26251 | -0.97 | 575.12; 865.20; 1001.21 |
| 71 | FO | Quercetin-diglucoside isomer | 10.58 | C_27_H_30_O_17_ | 626.14831 | 625.14048 | 625.14130 | -1.30 | 301.04; 463.09 |
| 72 |  | Unknown | 10.62 | C_16_H_20_O_10_ | 372.10565 | 371.09783 | 371.09827 | -1.19 | 121.03; 249.06 |
| 73 |  | Unknown | 10.68 | C_20_H_30_O_11_ | 446.17882 | 445.17099 | 445.17158 | -1.31 | 239.13; 283;12 |
| 74 | CT | Procyanidin pentamer | 10.70 | C_75_H_62_O_30_ | 1442.33260 | 1441.32478 | 1441.32746 | -1.86 | 720.16 [M-2H]^-2^ |
| 75 | CT | Procyanidin tetramer one A-type linkage | 10.72 | C_60_H_48_O_24_ | 1152.25356 | 1151.24574 | 1151.24694 | -1.04 | 289.07; 711.13; 863.11; |
| 76 | CM | Isopimpinellin pentoside | 10.86 | C_17_H_30_O_9_ | 378.18899 | 377.18116 | 377.18175 | -1.55 | 245.04 |
| 77 | FNO | Myricetin glucoside | 11.11 | C_21_H_20_O_13_ | 480.09040 | 479.08257 | 479.08291 | -0.70 | 316.02; 318.03 |
| 78 | PG | Darendroside B | 11.12 | C_21_H_32_O_12_ | 476.18938 | 475.18156 | 475.18226 | -1.47 | 101.02; 163.06; 205.07 |
| 79 |  | Unknown | 11.21 | C_18_H_22_O_9_ | 382.12639 | 381.11856 | 381.11915 | -1.53 | 233.08; 261.08 |
| 80 | CT | Procyanidin pentamer | 11.29 | C_75_H_62_O_30_ | 1442.33260 | 1441.32478 | 1441.32769 | -2.02 | 720.16 [M-2H]^-2^ |
| 81 |  | Unknown | 11.33 | C_19_H_18_O_10_ | 406.09000 | 405.08218 | 405.08272 | -1.33 | 229.05 |
| 82 | FO | Methyl dihydroquercetin hexoside | 11.41 | C_22_H_24_O_12_ | 480.12678 | 479.11896 | 479.11936 | -0.83 | 257.04; 316.02 |
| 83 |  | Unknown | 11.43 | C_18_H_32_O_10_ | 408.19955 | 407.19173 | 407.19243 | -1.72 | 101.02; 163.06; 205.07 |
| 84 | LN | Lariciresinol-glucoside | 11.53 | C_26_H_34_O_11_ | 522.21012 | 521.20229 | 521.20294 | -1.24 | 359.15 |
| 85 |  | Unknown | 11.56 | C_31_H_36_O_17_ | 680.19526 | 679.18743 | 679.18842 | -1.45 | 211.07; 323.09; 421.10; 633.18 |
| 86 | FG | Kaempferol-hexoside | 11.63 | C_21_H_20_O_11_ | 448.10057 | 447.09274 | 447.09333 | -1.31 | 285.04 |
| 87 | FNO | Dihydromyricetin | 11.73 | C_15_H_12_O_8_ | 320.05322 | 319.04540 | 319.04576 | -1.13 | 139.04; 183.03 |
| 88 | FO | Quercetin-glucosyl-xyloside isomer | 11.80 | C_26_H_28_O_16_ | 596.13774 | 595.12992 | 595.13080 | -1.48 | 301.04; 433.08 |
| 89 | FNO | Myricetin-arabinoside | 11.92 | C_20_H_18_O_12_ | 450.07983 | 449.07201 | 449.07267 | -1.47 | 316.02; 317.03 |
| 90 | CT | Procyanidin tetramer one A-type linkage | 12.08 | C_60_H_48_O_24_ | 1152.25356 | 1151.24574 | 1151.24782 | -1.81 | 289.07; 711.13; 863.11; |
| 91 | FO | Quercetin-rutinoside isomer | 12.38 | C_27_H_30_O_16_ | 610.15339 | 609.14557 | 609.14637 | -1.31 | 300.03; 447.09 |
| 92 | BG | Pentahydroxybenzophenone acetyl-xylopyranoside | 12.45 | C_20_H_20_O_11_ | 436.10057 | 435.09274 | 435.09328 | -1.23 | 243.03; 261.04 |
| 93 | FO | Quercetin-rutinoside isomer | 12.49 | C_27_H_30_O_16_ | 610.15339 | 609.14557 | 609.14633 | -1.25 | 301.04 |
| 94 | CM | Methoxycoumarin-acetic acid | 12.56 | C_12_H_10_O_5_ | 234.05283 | 233.04500 | 233.04490 | 0.45 | 189.05 |
| 95 | FN | Isovitexin | 12.58 | C_21_H_20_O_10_ | 432.10565 | 431.09783 | 431.09843 | -1.39 | 283.06; 311.06 |
| 96 | CT | Procyanidin trimer | 12.71 | C_45_H_38_O_18_ | 866.20582 | 865.19800 | 865.19904 | -1.20 | 287.06; 425.08; 577.14; 695.14 |
| 97 | CM | Urolithin c 3-sulfate | 12.81 | C_13_H_8_O_8_S | 323.99399 | 322.98617 | 322.98658 | -1.26 | 185.02; 243.03; 322.99 |
| 98 | FNO | Myricetin-arabinoside | 12.88 | C_20_H_18_O_12_ | 450.07983 | 449.07201 | 449.07258 | -1.27 | 316.02; 317.03 |
| 99 | CT | B-type procyanidin dimer | 12.91 | C_30_H_26_O_12_ | 578.14243 | 577.13461 | 577.13530 | -1.20 | 125.02; 289.07; 407.08 |
| 100 | PG | Petiolin F | 12.99 | C_19_H_20_O_10_ | 408.10565 | 407.09783 | 407.09842 | -1.45 | 257.05; 275.06 |
| 101 | FO | Taxifolin-rhamnoside | 13.02 | C_21_H_22_O_11_ | 450.11622 | 449.10839 | 449.10891 | -1.15 | 151.00; 287.05; 316.02 |
| 102 | FG | Kaempferol galactoside-rhamnoside | 13.06 | C_27_H_30_O_15_ | 594.15848 | 593.15065 | 593.15156 | -1.53 | 284.03; |
| 103 | FN | Phloridzin | 13.06 | C21H24O10 | 436.13695 | 435.12913 | 435.12974 | -1.40 | 227.07; 273.08 |
| 104 | CT | Procyanidin tetramer one A-type linkage | 13.09 | C_60_H_48_O_24_ | 1152.25356 | 1151.24574 | 1151.24774 | -1.74 | 289.07; 711.13; 863.11; |
| 105 | HBA | Ellagic acid | 13.15 | C_14_H_6_O_8_ | 302.00627 | 300.99845 | 300.99875 | -1.00 | 229.013; 257.01 |
| 106 | FO | Quercetin-hexoside | 13.25 | C_21_H_20_O_12_ | 464.09548 | 463.08766 | 463.08830 | -1.38 | 300.03; 301.03 |
| 107 |  | Unknown | 13.35 | C_20_H_30_O_11_ | 446.17882 | 445.17099 | 445.17164 | -1.45 | 239.14; 283.11 |
| 108 | FO | Quercetin glucosyl-xyloside isomer | 13.37 | C_26_H_28_O_16_ | 596.13774 | 595.12992 | 595.13050 | -0.97 | 301.04 |
| 109 | FG | Kaempferol-hexoside | 13.40 | C_21_H_20_O_11_ | 448.10057 | 447.09274 | 447.09342 | -1.51 | 285.04 |
| 110 | FO | Quercetin-hexoside | 13.49 | C_21_H_20_O_12_ | 464.09548 | 463.08766 | 463.08835 | -1.49 | 300.03; 301.03 |
| 111 | FG | Kaempferol/luteolin-rutinoside | 13.57 | C_27_H_30_O_15_ | 594.15848 | 593.15065 | 593.15140 | -1.26 | 284.03 |
| 112 | F-3-ol | Cinchonain I isomer | 13.59 | C_24_H_20_O_9_ | 452.11074 | 451.10291 | 451.10362 | -1.56 | 217.01; 341.07 |
| 113 |  | Unknown | 13.70 | C_19_H_24_O_9_ | 396.14204 | 395.13421 | 395.13468 | -1.18 | 247.10; 275.09 |
| 114 |  | Unknown | 13.86 | C_24_H_34_O_14_ | 546.19486 | 545.18704 | 545.18812 | -1.98 | 125.02; 219.07; 433.13; 475.14; |
| 115 | CT | A-type procyanidin dimer | 14.02 | C_30_H_24_O_12_ | 576.12678 | 575.11896 | 575.11986 | -1.56 | 125.02; 289.07; 407.08 |
| 116 |  | Unknown | 14.05 | C_20_H_32_O_9_ | 416.20464 | 415.19681 | 415.19752 | -1.70 | 167.03; 207.10; 253.11; 347.17 |
| 117 | FG | Dihydroferulic acid glucuronide | 14.12 | C_16_H_20_O_10_ | 372.10565 | 371.09783 | 371.09831 | -1.29 | 151.07; 196.07 |
| 118 | CT | Procyanidin tetramer | 14.14 | C_60_H_50_O_24_ | 1154.26921 | 1153.26139 | 1153.26251 | -0.97 | 575.12; 865.20; 983.20 |
| 119 | AGG | Regaloside A/H | 14.25 | C_18_H_24_O_10_ | 400.13695 | 399.12913 | 399.12980 | -1.68 | 125.02; 149.02; 237.08 |
| 120 | FO | Taxifolin-pentoside | 14.36 | C_20_H_20_O_11_ | 436.10057 | 435.09274 | 435.09360 | -1.97 | 151.00; 285.04; 301.03 |
| 121 |  | Unknown | 14.48 | C_22_H_32_O_13_ | 504.18430 | 503.17647 | 503.17729 | -1.62 | 209.08 |
| 122 | FG | Kaempferol-hexoside | 14.64 | C_21_H_20_O_11_ | 448.10057 | 447.09274 | 447.09317 | -0.95 | 284.04; 285.04 |
| 123 | FO | Quercetin-pentoside isomer | 14.77 | C_20_H_18_O_11_ | 434.08492 | 433.07709 | 433.07752 | -0.98 | 300.03; 301.04 |
| 124 | FO | Quercetin-pentoside isomer | 15.09 | C_20_H_18_O_11_ | 434.08492 | 433.07709 | 433.07755 | -1.05 | 300.03; 301.04 |
| 125 | FO | Querceti-pentoside isomer | 15.34 | C_20_H_18_O_11_ | 434.08492 | 433.07709 | 433.07746 | -0.84 | 300.03; 301.04 |
| 126 | FO | Quercetrin | 15.47 | C_21_H_20_O_11_ | 448.10057 | 447.09274 | 447.09332 | -1.29 | 300.03; 301.4 |
| 127 | FG | Kaempferol-pentoside | 16.22 | C_20_H_18_O_10_ | 418.09000 | 417.08218 | 417.08289 | -1.70 | 284.03; 285.04 |
| 128 |  | Oleuropein | 16.25 | C_25_H_32_O_13_ | 540.18430 | 539.17647 | 539.17730 | -1.53 | 275.09; 377.12; 395.13 |
| 129 | FO | Taxifolin-rhamnoside isomer | 16.26 | C_21_H_22_O_11_ | 450.11622 | 449.10839 | 449.10925 | -1.90 | 135.04; 151.03; 193.05; 287.05 |
| 130 | FO | Quercetin-acetyl-galactoside-rhamnoside | 16.42 | C_29_H_32_O_17_ | 652.16396 | 651.15613 | 651.15717 | -1.59 | 300.03 |
| 131 | XD | Kielcorin | 16.55 | C_24_H_20_O_8_ | 436.11582 | 435.10800 | 435.10866 | -1.52 | 217.01 |
| 132 | FO | Isorhamnetin-hexoside | 16.66 | C_22_H_22_O_12_ | 478.11113 | 477.10331 | 477.10392 | -1.28 | 314.04 |
| 133 | FO | Quercetin-acetyl-glucoside | 16.72 | C_23_H_22_O_13_ | 506.10605 | 505.09822 | 505.09900 | -1.53 | 178.99; 300.03; 463.09 |
| 134 | HBA | Phloretin-glucoside | 16.94 | C_21_H_24_O_10_ | 436.13695 | 435.12913 | 435.12996 | -1.91 | 167.03; 273.07 |
| 135 |  | Unknown | 17.14 | C_25_H_36_O_14_ | 560.21051 | 559.20269 | 559.20331 | -1.11 | 223.08; 433.14 |
| 136 | FG | Kaempferol-pentoside | 17.20 | C_20_H_18_O_10_ | 418.09000 | 417.08218 | 417.08283 | -1.56 | 284.03; 285.04 |
| 137 | FO | Quercetin-acetyl-glucoside | 17.31 | C_23_H_22_O_13_ | 506.10605 | 505.09822 | 505.09900 | -1.53 | 300.03; 463.07 |
| 138 | FNO | Myricetin | 17.54 | C_15_H_10_O_8_ | 318.03757 | 317.02975 | 317.03006 | -0.98 | 137.03; 151.00; 179.00 |
| 139 | FG | Kaempferol-rhamnoside | 17.68 | C_21_H_20_O_10_ | 432.10565 | 431.09783 | 431.09840 | -1.32 | 285.04; 317.03 |
| 140 | FG | Chrysoeriol-glucoside | 17.78 | C_22_H_22_O_11_ | 462.11622 | 461.10839 | 461.10929 | -1.94 | 298.05 |
| 141 | PD | Hyperielliptone HC | 17.89 | C_25_H_22_O_10_ | 482.12130 | 481.11348 | 481.11439 | -1.89 | 341.07 |
| 142 | FO | Taxifolin-rhamnoside isomer | 17.91 | C_21_H_22_O_11_ | 450.11622 | 449.10839 | 449.10928 | -1.97 | 257.05; 275.06 |
| 143 | F-3-ol | Cinchonain I isomer | 18.22 | C_24_H_20_O_9_ | 452.11074 | 451.10291 | 451.10372 | -1.78 | 217.01; 341.07 |
| 144 |  | Unknown | 18.39 | C_25_H_34_O_13_ | 542.19995 | 541.19212 | 541.19299 | -1.60 | 233.08 |
| 145 | AQ | Skyrin glucoside | 18.65 | C_36_H_28_O_15_ | 700.14283 | 699.13500 | 699.13614 | -1.62 | 151.00; 443.04; 537.08 |
| 146 |  | Unknown | 18.72 | C_10_H_16_O_4_ | 200.10486 | 199.09704 | 199.09669 | 1.76 | 114.95 |
| 147 |  | Unknown | 18.76 | C_22_H_38_O_9_ | 446.25159 | 445.24376 | 445.24457 | -1.81 | 285.04; 314.04; 315.06 |
| 148 | FG | Kaempferol-acetyl-glucoside | 18.97 | C_23_H_22_O_12_ | 490.11113 | 489.10331 | 489.10422 | -1.86 | 284.03 |
| 149 |  | Unknown | 19.10 | C_23_H_36_O_13_ | 520.21560 | 519.20777 | 519.20875 | -1.88 | 165.09; 487.25 |
| 150 | FG | Chrysoeriol-glucuronide | 19.38 | C_22_H_20_O_12_ | 476.09548 | 475.08766 | 475.08835 | -1.45 | 300.03 |
| 151 |  | Unknown | 19.47 | C_26_H_24_O_12_ | 528.12678 | 527.11896 | 527.11957 | -1.16 | 243.03 |
| 152 | FN | Apigenin hexoside | 21.00 | C_21_H_20_O_10_ | 432.10565 | 431.09783 | 431.09839 | -1.30 | 269.04; 298.05 |
| 153 | FN | Luteolin | 21.84 | C_15_H_10_O_6_ | 286.04774 | 285.03992 | 285.04034 | -1.47 | 133.02; 151.00 |
| 154 | FO | Quercetin | 22.03 | C_15_H_10_O_7_ | 302.04266 | 301.03483 | 301.03518 | -1.15 | 107.01; 151.00 |
| 155 | FA | Trihydroxy octadecadienoic acid isomer | 24.02 | C_18_H_32_O_5_ | 328.22498 | 327.21715 | 327.21760 | -1.36 | 171.10;211.13; 229.14 |
| 156 | FA | Trihydroxy octadecadienoic acid isomer | 24.25 | C_18_H_32_O_5_ | 328.22498 | 327.21715 | 327.21761 | -1.39 | 171.10;211.13; 229.14 |
| 157 | FA | Trihydroxy octadecadienoic acid isomer | 24.48 | C_18_H_32_O_5_ | 328.22498 | 327.21715 | 327.21760 | -1.36 | 171.10;211.13; 229.14 |
| 158 | XD | Trihydroxyxanthone isomer | 24.98 | C_13_H_8_O_5_ | 244.03718 | 243.02935 | 243.02947 | -0.47 | 143.05; 171.04; 199.04; |
| 159 | FA | Pinellic acid | 26.29 | C_18_H_34_O_5_ | 330.24063 | 329.23280 | 329.23327 | -1.41 | 211.13 |
| 160 | FN | Biapigenin isomer | 27.38 | C_30_H_18_O_10_ | 538.09000 | 537.08218 | 537.08257 | -0.73 | 151.00; 385.07; 443.04 |
| 161 | PQ | Diethylene glycol monolaurate | 28.00 | C_16_H_32_O_4_ | 288.23006 | 287.22224 | 287.22267 | -1.50 | 99.08; 141.13; 269.21 |
| 162 | FN | Biapigenin isomer | 28.41 | C_30_H_18_O_10_ | 538.09000 | 537.08218 | 537.08258 | -0.74 | 375.05; 417.06; 443.04 |
| 163 |  | Unknown | 28.42 | C_15_H_14_O_7_ | 306.07396 | 305.06613 | 305.06666 | -1.72 | 123.01; 246.05 |
| 164 | FN | Biapigenin isomer | 29.10 | C_30_H_18_O_10_ | 538.09000 | 537.08218 | 537.08259 | -0.76 | 385.07 |
| 165 | HCA | Hexyl salicylate | 29.14 | C_13_H_18_O_3_ | 222.12560 | 221.11777 | 221.11762 | 0.70 | 109.06 |
| 166 | FAL | Dodecyl sulfate | 29.77 | C_12_H_26_O_4_S | 266.15518 | 265.14736 | 265.14775 | -1.46 | 96.96 |
| 167 | PD | Geranyl phlorisobutyrophenone | 30.47 | C_20_H_28_O_4_ | 332.19876 | 331.19094 | 331.19131 | -1.12 | 268.09; 287.2 |
| 168 |  | Unknown | 30.66 | C_25_H_48_O_11_S | 556.29174 | 555.28391 | 555.28450 | -1.05 | 225.01 |
| 169 | BD | Dodecylbenzenesulfonic acid | 30.86 | C_18_H_30_O_3_S | 326.19157 | 325.18374 | 325.18419 | -1.36 | 183.01 |
| 170 | AQ | Protopseudohypericin | 31.39 | C_30_H_18_O_9_ | 522.09509 | 521.08726 | 521.08783 | -1.08 | 152.99; 521.09 |
| 171 | FN | Apigenin | 31.48 | C_15_H_10_O_5_ | 270.05283 | 269.04500 | 269.04539 | -1.43 | 225.06; 241.05 |
| 172 |  | Unknown | 31.80 | C_23_H_34_O_6_ | 406.23554 | 405.22772 | 405.22816 | -1.09 | 194.06; 209.08; 245.12 |
| 173 | AQ | Pseudohypericin | 31.82 | C_30_H_16_O_9_ | 520.07944 | 519.07161 | 519.07203 | -0.80 | 519.07 |
| 174 |  | Unknown | 31.93 | C_22_H_30_O_6_ | 390.20424 | 389.19642 | 389.19700 | -1.49 | 371.19 |
| 175 |  | Unknown | 32.14 | C_23_H_30_O_7_ | 418.19916 | 417.19133 | 417.19208 | -1.79 | 249.08; 373.20 |
| 176 |  | Unknown | 32.25 | C_25_H_36_O_6_ | 432.25119 | 431.24337 | 431.24381 | -1.02 | 209.12; 249.11; 277.18; 317.18 |
| 177 | PD | Olympicin C/E | 32.30 | C_21_H_30_O_5_ | 362.20933 | 361.20150 | 361.20212 | -1.70 | 221.08; 235.10 |
| 178 |  | Unknown | 32.32 | C_25_H_36_O_6_ | 432.25119 | 431.24337 | 431.24381 | -1.02 | 209.12; 249.11; 277.18; 317.18 |
| 179 |  | Unknown | 32.35 | C_21_H_30_O_3_ | 330.21950 | 329.21167 | 329.21228 | -1.84 | - |
| 180 |  | Unknown | 32.41 | C_25_H_36_O_6_ | 432.25119 | 431.24337 | 431.24381 | -1.02 | 209.12; 249.11; 277.18; 317.18 |
| 181 |  | Unknown | 32.56 | C_30_H_20_O_9_ | 524.11074 | 523.10291 | 523.10347 | -1.06 | 269.05 |
| 182 |  | Unknown | 32.62 | C_25_H_36_O_5_ | 416.25628 | 415.24845 | 415.24897 | -1.24 | 331.19; 345.21 |
| 183 |  | Unknown | 32.62 | C_45_H_64_O_9_ | 748.45504 | 747.44721 | 747.44831 | -1.46 | 415.25 |
| 184 | DT | Carnosic acid | 32.68 | C_20_H_28_O_4_ | 332.19876 | 331.19094 | 331.19148 | -1.63 | 151.08; 194.06; 207.07; 287.2 |
| 185 |  | Unknown | 32.69 | C_25_H_36_O_6_ | 432.25119 | 431.24337 | 431.24383 | -1.07 | 209.12; 249.1; 277.18; 317.17 |
| 186 | PD | Hypercalin A/Spirohypatone A/Hyphenol B | 32.89 | C_26_H_38_O_5_ | 430.27193 | 429.26410 | 429.26450 | -0.92 | 345.21 |
| 187 | PD | Chipericumin C/Hyperpatulone A/Hyphenol F | 32.96 | C_26_H_38_O_6_ | 446.26684 | 445.25902 | 445.25956 | -1.21 | 291.20; 331.19 |
| 188 | PD | Hyperatomanin/Hypercalyxone A | 33.01 | C_25_H_36_O_4_ | 400.26136 | 399.25354 | 399.25397 | -1.08 | 261.22 |
| 189 | DT | Methoxy-carnosic acid | 33.05 | C_21_H_30_O_4_ | 346.21441 | 345.20659 | 345.20695 | -1.04 | 221.08; 301.22 |
| 190 | PD | Chipericumin C/Hyperpatulone A/Hyphenol F | 33.13 | C_26_H_38_O_6_ | 446.26684 | 445.25902 | 445.25956 | -1.21 | 223.13; 263.13 |
| 191 | PAH | Ialibinone B/Hyperguinone B/Erectquione A | 33.14 | C_21_H_28_O_4_ | 344.19876 | 343.19094 | 343.19135 | -1.19 | 203.07; 315.20 |
| 192 | PD | 3-Geranyl-1-(2-methylpropanoyl)-phloroglucinol/Hyperjovinol B | 33.15 | C_20_H_28_O_4_ | 332.19876 | 331.19094 | 331.19148 | -1.63 | 151.08; 194.06; 288.2 |
| 193 | BD | Hyperpapuanone | 33.30 | C_26_H_38_O_4_ | 414.27701 | 413.26919 | 413.26969 | -1.21 | 261.22; 345.28 |
| 194 | PD | Olympicin C/E /Petiolin J | 33.43 | C_21_H_30_O_5_ | 362.20933 | 361.20150 | 361.20213 | -1.73 | 224.07 |
| 195 |  | Unknown | 33.45 | C_23_H_34_O_5_ | 390.24063 | 389.23280 | 389.23341 | -1.55 | 237.08; 261.15; 305.14 |
| 196 | PD | Pyramidatone A/Chipericumin C/Hyperpatulone A/Hyphenol F | 33.49 | C_26_H_38_O_6_ | 446.26684 | 445.25902 | 445.25956 | -1.21 | 238.08 |
| 197 | PD | 3-Geranyl-1-(2-methylbutanoyl)-phloroglucinol/Olympicin A | 33.51 | C_21_H_30_O_4_ | 346.21441 | 345.20659 | 345.20697 | -1.10 | 152.01; 208.07 |
| 198 | PAH | Hyperguinone A | 33.55 | C_20_H_26_O_4_ | 330.18311 | 329.17529 | 329.17582 | -1.61 | 245.08; 259.10 |
| 199 | PD | Bellumone R | 33.69 | C_27_H_40_O_4_ | 428.29266 | 427.28484 | 427.28531 | -1.10 | 383.3 |
| 200 |  | Unknown | 33.71 | C_25_H_36_O_6_ | 432.25119 | 431.24337 | 431.24381 | -1.02 | 249.1; 317.17 |
| 201 | PD | Enaimeone C/1′-Hydroxyialibinone D/Bellumone J | 33.78 | C_22_H_32_O_5_ | 376.22498 | 375.21715 | 375.21763 | -1.27 | 238.08 |
| 202 |  | Unknown | 33.89 | C_21_H_28_O_5_ | 360.19368 | 359.18585 | 359.18641 | -1.55 | 331.19 |
| 203 | PD | Enaimeone C/1′-Hydroxyialibinone D/Bellumone J | 33.91 | C_22_H_32_O_5_ | 376.22498 | 375.21715 | 375.21763 | -1.27 | 197.8; 288.14; 357.21 |
| 204 | BG | 4-Geranyloxy-2-hydroxy-6-isoprenyloxy- benzophenone | 33.96 | C_28_H_34_O_4_ | 434.24571 | 433.23789 | 433.23820 | -0.72 | 145.03; 281.08; 373.17 |
| 205 | PD | Hyperatomanin/Hypercalyxone A | 33.98 | C_25_H_36_O_4_ | 400.26136 | 399.25354 | 399.25398 | -1.10 | 194.05; 261.11 |
| 206 | PD | Hyperielliptone HB/Hyperpatulone B | 33.99 | C_27_H_40_O_6_ | 460.28249 | 459.27467 | 459.27534 | -1.46 | 342.12; 357.21; 412.23 |
| 207 | PAH | Hyperguinone B | 34.01 | C_21_H_28_O_4_ | 344.19876 | 343.19094 | 343.19135 | -1.19 | 259.10; 273.11 |
| 208 | PD | Chipericumin B/Uralin D | 34.09 | C_27_H_40_O_7_ | 476.27741 | 475.26958 | 475.27024 | -1.38 | 251.13; 319.19 |
| 209 | PD | Chipericumin C/Hyperpatulone A/Hyphenol F | 34.17 | C_26_H_38_O_6_ | 446.26684 | 445.25902 | 445.25956 | -1.21 | 263.13; 331.19 |
| 210 |  | Unknown | 34.18 | C_23_H_30_O_7_ | 418.19916 | 417.19133 | 417.19208 | -1.79 | 357.17 |
| 211 | AQ | Protohypericin | 34.32 | C_30_H_18_O_8_ | 506.10017 | 505.09235 | 505.09312 | -1.52 | 399.26; 505.09 |
| 212 |  | Unknown | 34.36 | C28H42O6 | 474.29814 | 473.29032 | 473.29096 | -1.35 | 357.21;425.24 |
| 213 | ST | Hyperevolutin A/Prolifenone B | 34.54 | C_30_H_44_O_4_ | 468.32396 | 467.31614 | 467.31662 | -1.03 | 231.14; 275.13; 423.33 |
| 214 |  | Unknown | 34.72 | C25H36O5 | 416.25628 | 415.24845 | 415.24897 | -1.24 | 235.06; 278.12; 345.18 |
| 215 |  | Unknown | 34.77 | C16H32O3 | 272.23515 | 271.22732 | 271.22765 | -1.20 | 225.22 |
| 216 |  | Unknown | 34.81 | C24H32O7 | 432.21481 | 431.20698 | 431.20728 | -0.68 | 371.19 |
| 217 | BD | Papuaforin B/Hyperpapuanone | 34.96 | C_26_H_38_O_4_ | 414.27701 | 413.26919 | 413.26969 | -1.21 | 233.08; 275.13 |
| 218 | PD | Hyperpolyphyllirin/Prolifenone A/Hyphenol B/Bellumone B | 35.11 | C_31_H_46_O_4_ | 482.33961 | 481.33179 | 481.33233 | -1.12 | 289.14; 438.35 |
| 219 | PD | Chinesin II/Hypercalin A/Spirohypatone A/Hyphenol B | 35.37 | C_26_H_38_O_5_ | 430.27193 | 429.26410 | 429.26450 | -0.92 | 249.08; 292.13; 360.19 |
| 220 |  | Unknown | 35.37 | C25H36O4 | 400.26136 | 399.25354 | 399.25396 | -1.05 | 111.04 |
| 221 | PD | Bellumone R | 35.69 | C_27_H_40_O_4_ | 428.29266 | 427.28484 | 427.28525 | -0.96 | 289.14 |
| 222 | BD | Hyperpapuanone/Papuaforin B | 35.71 | C_26_H_38_O_4_ | 414.27701 | 413.26919 | 413.26969 | -1.21 | 275.13 |
| 223 | BD | Crassipin H | 35.79 | C_42_H_58_O_9_ | 706.40809 | 705.40026 | 705.40137 | -1.57 | 233.08; 345.21; 359.19 |
| 224 |  | Unknown | 35.87 | C44H62O10 | 750.43430 | 749.42648 | 749.42755 | -1.43 | 319.19 |
| 225 | BD | Hyperpapuanone/Papuaforin B | 36.12 | C_26_H_38_O_4_ | 414.27701 | 413.26919 | 413.26969 | -1.21 | 125.05; 261.11; 329.21 |
| 226 | PD | Bellumone R | 36.54 | C_27_H_40_O_4_ | 428.29266 | 427.28484 | 427.28531 | -1.10 | 289.14 |
| 227 | PD | Hyperfirin | 36.88 | C_30_H_44_O_4_ | 468.32396 | 467.31614 | 467.31672 | -1.24 | 287.13; 329.18 |
| 228 | BD | Crassipin C | 36.99 | C_42_H_56_O_8_ | 688.39752 | 687.38970 | 687.39069 | -1.44 | 385.2; 550.26; 643.40 |
| 229 | PD | Bellumone R | 37.05 | C_27_H_40_O_4_ | 428.29266 | 427.28484 | 427.28531 | -1.10 | 125.06; 275.13; 343.23 |
| 230 |  | Unknown | 37.37 | C43H58O9 | 718.40809 | 717.40026 | 717.40131 | -1.46 | 345.21;642.39 |
| 231 | PD | Hyperbrasilol C | 38.02 | C_32_H_42_O_8_ | 554.28797 | 553.28015 | 553.28089 | -1.34 | 221.08; 343.19 |
| 232 | PD | Adhyperfirin | 38.06 | C_31_H_46_O_4_ | 482.33961 | 481.33179 | 481.33234 | -1.14 | 301.14; 343.19 |
| 233 | PD | Hyperibone J | 38.20 | C_31_H_46_O_5_ | 498.33453 | 497.32670 | 497.32728 | -1.16 | 301.14; 331.19; 413.27 |
| 234 |  | Unknown | 38.40 | C_28_H_36_O_5_ | 452.25628 | 451.24845 | 451.24921 | -1.67 | 327.12; 382.18 |
| 235 |  | Unknown | 38.64 | C_33_H_46_O_5_ | 522.33453 | 521.32670 | 521.32728 | -1.10 | 384.19 |

*HBA- hydroxybenzoic acids and derivatives; HCA - hydroxycinnamic acids and derivatives; F-3-ol - Flavan-3-ols; HT - hydrolysable tannins and derivatives; CT - condensed tannins and derivatives; IF - isoflavones; FNN-flavanones, FO - flavonols; FN-flavones; LN-lignans; FNO-flavanonols; AQ- anthraquinones and derivatives; CM-coumarins and derivatives; FA -fatty acids and derivatives; CA - Cycloalkane (Napthene), COA - carboxylic acids, AGG - acylated glycerol glucosides, PG - phenolic glucosides, XD - Xanthone derivatives, PD - Phloroglucinol derivatives, PQ - Perylenequinones, BD – benzene derivatives, ST – Sesquiterpenoids, PAH - Polycyclic aromatic hydrocarbon, DT – Diterpenoids, PC - phenolic glycosides, BG - benzophenone glycosides, FG – flavonoid glycoside, FAL - Fatty alcohols
